# Systematic validation of BACH1 antibodies enables the identification of BACH1 localisation as predictor of prognosis in early-stage lung cancer

**DOI:** 10.1016/j.redox.2026.104432

**Published:** 2026-10-08

**Authors:** Yumna Sharaf, Donika Klenja-Skudrinja, Maureen Higgins, Susan Bray, David Walker, Hazem Abdullah, In Hwa Um, Aileen Sandilands, Neil Kernohan, Albena T. Dinkova-Kostova, David J. Harrison, Laureano de la Vega

**Affiliations:** a Jacqui Wood Cancer Centre, Division of Cancer Research, School of Medicine, University of Dundee, UK; b School of Medicine, University of Dundee, UK; c School of Medicine, University of St Andrews, UK; d NHS, Ninewells Hospital, Dundee, UK

## Abstract

The transcription factor BACH1 is a key regulator of cellular stress responses, metabolism, and tumour progression, and has emerged as a promising therapeutic target in several diseases, particularly metastatic cancer, inflammatory disease and cardiovascular conditions. As interest in BACH1 biology and its clinical relevance grows, the field would benefit from robust, well-characterised reagents enabling reliable detection of BACH1 across diverse experimental platforms, including analysis of human tissue. Establishing such tools is essential for advancing mechanistic studies and recognising the potential of BACH1 as a prognostic biomarker.

We performed the first systematic validation of commercially available antibodies against human BACH1 across multiple experimental platforms, including western blotting, immunohistochemistry, and multiplex immunofluorescence. To ensure robustness, we validated antibodies using complementary approaches, including BACH1-knockout and knockdown cell lines, pharmacological modulators of BACH1 stability and localisation, and multiple extraction conditions. Their performance in immunohistochemistry was further tested using fixed cell pellets from wild-type and BACH1-deficient cells. Validated antibodies were subsequently applied to normal human tissues and lung cancer tissue microarrays to investigate BACH1 expression and subcellular localisation, and quantitative multiplex single-cell immunofluorescence analysis was performed in a cohort of early-stage lung adenocarcinoma patients with clinical follow-up. We demonstrate that subcellular localisation of BACH1 in early-stage lung adenocarcinoma correlates with patient prognosis, underscoring its clinical and translational significance.

Our study delivers a rigorously validated toolkit for the BACH1 research community, removes a major barrier to progress in the field, and highlights the potential of BACH1 localisation as a biomarker in early-stage lung adenocarcinoma.

## 1 Introduction

BTB and CNC homology 1 (BACH1) is a transcription factor of the Cap ‘n’ Collar basic region leucine zipper (CNC-bZIP) family that primarily acts as a transcriptional repressor of genes involved in oxidative stress responses and heme metabolism, making BACH1 a key modulator of cellular adaptation to stress [[1], [2], [3]].

Evidence from BACH1-deficient mouse models highlights its physiological relevance: although BACH1-knockout mice are viable and grossly normal, they exhibit impaired muscle regeneration [4] and show remarkable resistance to a variety of stress conditions, including oxidative and inflammatory insults, ischemia-reperfusion injury, and vascular remodelling [[5], [6], [7], [8], [9], [10], [11]]. These observations suggest that loss of BACH1 confers broad protection against tissue damage and dysfunction, likely through sustained activation of antioxidant and metabolic defence pathways. Accordingly, BACH1 has been implicated in cardiovascular disease, atherosclerosis, neurodegeneration, and metabolic disorders [9,[12], [13], [14]].

In oncology, BACH1 has emerged as a key regulator of tumour metastasis. Across multiple tumour types, including lung and breast tumours, BACH1 promotes metabolic reprogramming and invasive behaviour [15,16]. Importantly, elevated BACH1 expression has been associated with poor clinical outcomes in several malignancies, including lung adenocarcinoma, triple-negative breast cancer, and pancreatic ductal adenocarcinoma [[15], [16], [17]], highlighting its dual potential as a therapeutic target and a prognostic biomarker particularly in lung cancer. We have recently identified a BACH1-associated transcriptional signature in lung cancer [18]; however, this signature has not been validated in tissue, and thus direct assessment of BACH1 protein levels and localisation in tumour samples is still essential to determine its functional and prognostic roles.

Despite the broad disease relevance of BACH1 and the rapid growth of this field, further progress, particularly in establishing BACH1 as a prognostic biomarker, will benefit from the development of well-validated reagents that enable consistent detection across platforms. While antibody-based approaches remain important, questions around specificity, sensitivity, and reproducibility can affect confidence in currently available tools. For example, BACH1 has been reported at varying molecular weights in western blots, often without clear validation of band identity. In addition, although previous studies have examined BACH1 levels in tissue samples [[19], [20], [21]], more thoroughly validated protocols would strengthen these findings, as some published staining data lack appropriate controls or sufficient resolution to define subcellular localisation. Overall, the continued refinement and standardisation of methods for accurately detecting BACH1 in patient tissues will be an important step forward.

Here, we present the first systematic and comprehensive validation of a panel of anti-BACH1 antibodies for use in western blotting (WB), immunoprecipitation (IP), tissue immunohistochemistry (IHC) and tissue multiplexed immunofluorescence (mIF). To ensure robustness, we employed complementary strategies including BACH1-knockout and -knockdown cell lines, pharmacological modulators with distinct mechanisms of action, and a panel of lung cancer cell lines. Antibodies demonstrating high specificity and reproducibility were then used in optimised immunohistochemistry protocols across a broad range of normal human tissues and lung cancer tissue microarrays and in multiplex immunofluorescence protocols in early-stage lung adenocarcinoma tissue microarrays. These analyses enabled us to assess the clinical significance of BACH1 subcellular localisation in lung cancer patient samples. Collectively, our study provides a rigorously validated toolkit for the BACH1 research community and establishes BACH1 localisation as a promising prognostic biomarker in early-stage lung adenocarcinoma.

## 2 Material & methods

### 2.1 Cell culture

Cells were cultured in Dulbecco's Modified Eagle Medium (DMEM) at 37°C and 5% CO2. Media were obtained from Thermo Fisher Scientific and supplemented with 10% FBS. All cells were either validated by Short Tandem Repeat (STR) profiling or obtained from ATCC and were routinely tested for mycoplasma. CRISPR-edited BACH1-KO cells were produced as previously described [22,23]. Control cells, referred to as wild type (WT) are the pooled population of surviving cells transfected with an empty pLentiCRISPRv2 vector treated with puromycin.

### 2.2 Reagents and antibodies

Information for all the BACH1 antibodies is provided in Fig. 1a. Antibody against ALPHA-TUBULIN was obtained from Sigma-Aldrich (T5168), anti-LAMIN B2 (C-20) from Santa Cruz Biotechnology, anti-NRF2 antibody (D1Z9C) was obtained from Cell Signalling Technology and anti-HMOX1 antibody was purchased from Abcam (ab52947). Horseradish peroxidase (HRP)-conjugated secondary antibodies were from Life Technologies.

**Fig. 1:**
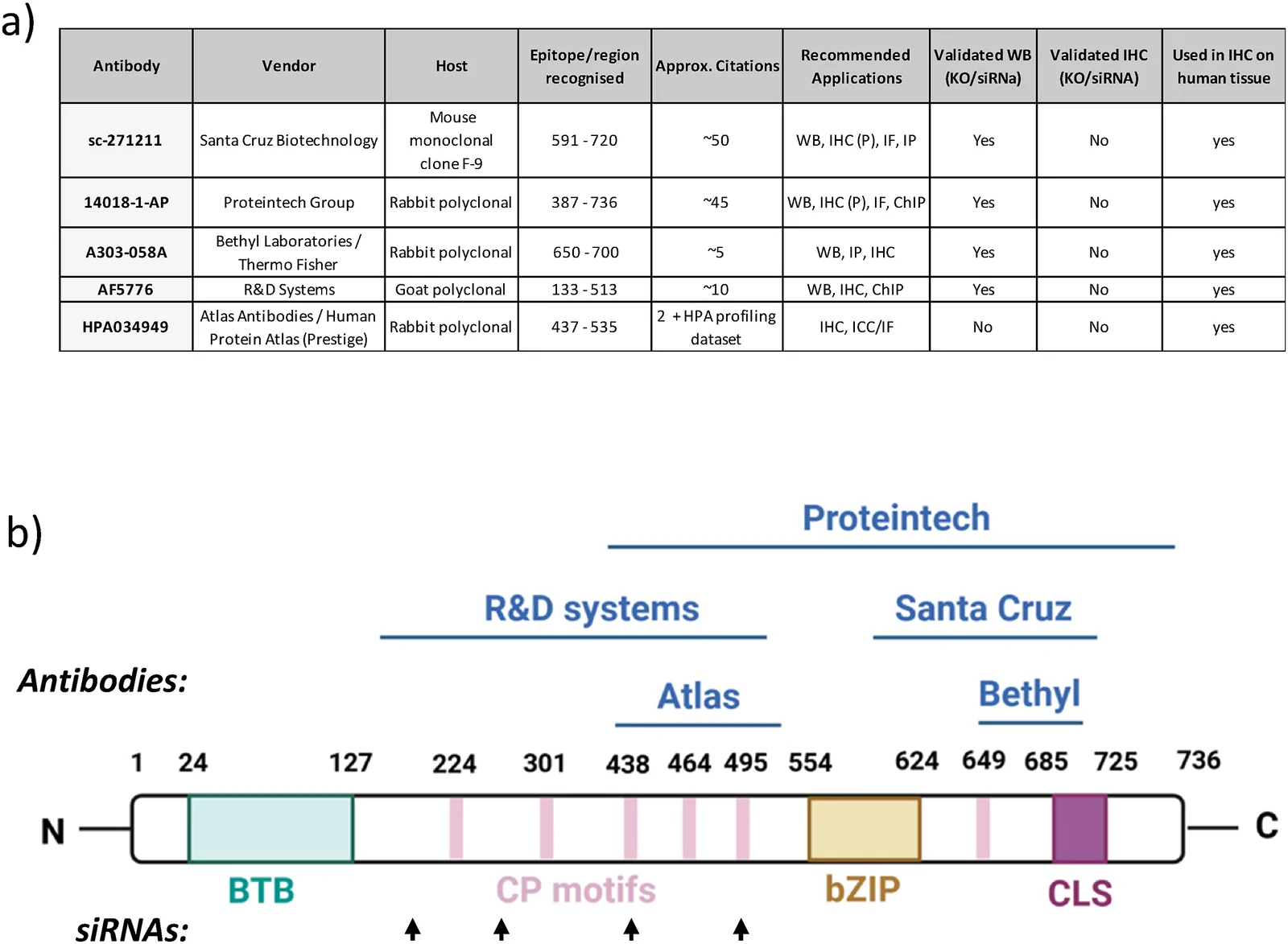
**Overview of BACH1 reagents**. **a)** Summary table of key information for the antibodies used in this study. **b)** Schematic representation of the BACH1 protein, indicating antibodies and siRNA target regions and domain organisation.

siRNAs for BACH1 were ON-TARGETplus SMARTpool siRNAs obtained from Horizon Discovery. Hemin and CDDO-TFEA were obtained from Cayman Chemicals.

### 2.3 Western blotting and subcellular fractionation

Cells were washed and harvested in ice-cold phosphate-buffered saline (PBS). For whole-cell extracts, cells were lysed in RIPA buffer supplemented with phosphatase and protease inhibitors. Lysates were sonicated for 20 s at 20% amplitude and then subjected to centrifugation for 10 min at 4°C. For subcellular fractionation, cells were resuspended in 300 μl of low-salt buffer A (10 mM HEPES/KOH pH7.9, 10 mM KCl, 0.1 mM EDTA, 0.1 mM EGTA, 1 mM β-mercaptoethanol) and incubated for 10 min on ice before 10 μl of 10% NP-40 was added followed by gentle vortexing. The homogenate was subjected to centrifugation for 1 min at 13,000 rpm in a microcentrifuge, and the supernatant representing the cytoplasmic fraction was collected. The pellet containing the cell nuclei was washed four additional times in buffer A then resuspended in 100 μl high-salt buffer B (20 mM Hepes/KOH pH 7.9, 400 mM NaCl, 1 mM EDTA, 1 mM EGTA, 1 mM β-mercaptoethanol). The resuspended fractions were sonicated followed by centrifugation at 4°C for 10 min at 13,000 rpm. The supernatant representing the nuclear fraction was then collected and saved. The protein concentration in the whole-cell lysate, nuclear and cytoplasmic fractions were determined using the BCA assay (Thermo Fisher Scientific). The lysates were then mixed with SDS sample buffer and boiled for 5 min at 95°C. Equal amounts of protein from lysates were separated by SDS-PAGE, using either 8% or 10% Tris-Glycine gels, followed by semidry blotting to a polyvinylidene difluoride membrane (PVDF) (Thermo Fisher Scientific). Subsequently, the membrane was blocked with 5% (w/v) non-fat dried milk dissolved in Tris-buffered saline (TBS) with 0.1% v/v Tween-20 (TBST), followed by incubation with primary antibodies overnight at 4^°^C. The next day, the membranes were washed with TBST and appropriate secondary antibodies coupled to HRP were added and incubated for 1 h at RT. Following another set of TBST washes, the bound secondary antibodies were detected by enhanced chemiluminescence using ClarityTM Western ECL Blotting Substrate (Bio-Rad). The same protein ladder (PageRuler™ Prestained Protein Ladder, 10 to 180 kDa from Thermo) was used in all Western blot gels. Marker band positions were assigned according to the molecular weights specified by the manufacturer for the corresponding gel system, gel percentage, and running buffer. The apparent molecular weight of BACH1 was subsequently estimated by comparison with the appropriately assigned marker bands.

### 2.4 siRNA cell transfections

On the day prior to transfection, cells were plated to the required cell density (70-90% confluency). Lipofectamine RNAiMAX (Invitrogen) was used to introduce the siRNA. The siRNA and lipofectamine were individually diluted in OptiMEM (Gibco) and incubated for 10 min at RT. Diluted siRNAs were added to the diluted Lipofectamine solution (1:1 ratio) and further incubated for 15 min. The siRNA-lipid complexes were added to the cells and incubated 48 h in a humidified incubator at 37°C and 5% CO_2_ before harvesting.

### 2.5 Immunoprecipitations

Cells were washed and harvested in ice-cold phosphate-buffered saline (PBS) and lysed in IP buffer (50 mM Hepes pH 7.5, 50 mM NaCl, 1% (v/v) Triton X-100, 2 mM EDTA, 10 mM sodium fluoride, 0.5 mM sodium orthovanadate, leupeptin (10 μg/ml), aprotinin (10 μg/ml). Lysates were sonicated for 15 s at 20% amplitude and then cleared by centrifugation for 15 min at 4°C. 10% of the total volume was used as an INPUT material. The pull-down was performed with 1 μg of antibody plus 20 μl of Dynabeads™ Protein G for 30 min on a spinning wheel at 4°C. The beads were washed 3x with IP buffer and eluted by boiling in 1 x SDS sample buffer.

### 2.6 DAKO EnVision Flex IHC

Sections from formalin fixed paraffin embedded blocks (nominally 4 μm thick) were cut onto superfrost® plus slides (VWR International Ltd) and dried for 1 h at 60°C. Antigen retrieval and de-paraffinisation was performed using DAKO EnVision™ FLEX Target Retrieval solution (high pH) buffer (50x conc) (K8004) in a DAKO PT Link for 20 min at 97°C. Immunostaining using DAKO EnVision™ FLEX system was performed using a DAKO Link Autostainer. Sections were initially washed in Flex Wash Buffer (K8006) for 5 min at room temperature. The following steps were then performed manually: 1) Flex Peroxidase-Blocking Reagent (SM801) applied for 5 min at room temperature; 2) Incubation with primary antibody (diluted in DAKO EnVision™ FLEX antibody diluent. Santa Cruz, 1:50 dilution + secondary mouse linker, low pH retrieval. Protein Atlas, 1:200 dilution + secondary rabbit linker, high pH retrieval) overnight at 4°C. The following steps were then performed using the automated platform: 3) Flex/HRP labelled polymer (SM802) for 20 min; 4) Flex DAB + working solution (SM803) for 2 × 5 min; 5) Copper Sulphate solution for 5 min; 6) Flex Haematoxylin for 5 min. Between each step, sections were rinsed with Flex Wash Buffer with a final wash in dH_2_O. Sections known to stain positively were included in each batch of staining and negative controls were prepared by replacing the primary antibody with DAKO antibody diluent. Slides were manually washed in tap water before being rinsed in graded concentrations of alcohol, with 3 final rinses in Xylene. Glass coverslips were applied.

### 2.7 Commercial lung cancer tissue microarray for protocol validation

The lung cancer tissue microarray used to test the protocol was obtained from Tissuearray (Lc814a).

### 2.8 Patient cohort and tissue microarray construction for prognostic studies

Tumour samples were obtained from patients with early-stage non–small cell lung cancer (NSCLC) who underwent primary surgical resection at the Royal Infirmary of Edinburgh under NHS Research Scotland Lothian Bioresource approval (Lothian 10/S1402/33). The patient cohort has been previously described [24]. Eligible cases included patients with stage I–IIA/IIB lung adenocarcinoma, classified according to the Seventh Edition of the International Association for the Study of Lung Cancer (IASLC) staging system. Inclusion criteria required complete surgical resection without neoadjuvant chemotherapy or radiotherapy and no adjuvant treatment within 12 weeks of surgery or during the first month of follow-up. Patients with a previous history of lung cancer or synchronous primary tumours were excluded. Overall survival was determined from clinical records. A total of 174 cases were included based on the availability of formalin-fixed paraffin-embedded (FFPE) tissue and sufficient remaining material for analysis. All samples were anonymised prior to analysis, and laboratory investigations were performed blinded to clinical and pathological information. A five-year survival cut-off was applied for outcome analyses.

Tissue microarrays (TMAs) were constructed from representative tumour regions selected by a consultant pathologist. Eight tumour cores were obtained from each patient and incorporated into the TMA design to maximise representation of the tumour and account for intratumoural heterogeneity. The use of multiple cores enabled a more robust assessment of biomarker expression across different tumour regions and reduced the potential impact of regional variation in protein expression.

### 2.9 Clinicopathological characteristics of the patient cohort

174 patients with early-stage lung adenocarcinoma were included in the study. The median age at diagnosis was 68 ± 10 years, with 39% males and 61% females. Tumour size was <3 cm in 43% and ≥3 cm in 57% of cases. According to the IASLC staging system, 20% were stage IA, 30% stage IB, and 50% stage IIA/IIB. Pleural invasion was present in 36% and absent in 64% of patients. At five-year follow-up, 34% of patients had died from disease-related causes, while 66% were alive.

### 2.10 Multiplex immunofluorescence (mIF)

Multiplex immunofluorescence (mIF) staining was performed using the Leica Bond RX automated staining platform (Leica Biosystems). Formalin-fixed paraffin-embedded (FFPE) tissue blocks were sectioned at 3 μm thickness, mounted onto glass slides, and dried at 65°C. Automated deparaffinisation, rehydration, antigen retrieval, antibody incubation, and tyramide signal amplification (TSA) staining were performed on the Bond RX.

Antigen retrieval was carried out using Bond Epitope Retrieval Solution 1 (ER1, pH 6) at 100°C for 20 min. Endogenous peroxidase activity and non-specific protein binding were blocked using Bond Peroxide Block reagent for 5 min and casein serum-free protein block (Sigma, B6429, 500 ml, 1:10 dilution) for 10 min, respectively. A primary antibody against BACH1 (Human Protein Atlas, HPA034949; 1:250 dilution) was incubated for 40 min. Detection was achieved using an HRP-conjugated secondary antibody for 30 min followed by TSA Cyanine 3 fluorophore (Akoya Biosciences, NEL744001KT; 1:50 dilution) for 7 min. Following Tyramide-HRP covalent bond formation redundant antibodies that were not bound by covalent bond were stripped using ER1 at 95°C for 20 min to avoid cross contamination with the second primary visualisation.

Tumour epithelial cells were visualised using pan-cytokeratin AE1/AE3 (Agilent, M351501-1; 1:100 dilution). Detection was performed using a biotinylated anti-mouse secondary antibody (Thermo Fisher Scientific, 31800; 1:50), followed by streptavidin-conjugated Alexa Fluor 750 (Thermo Fisher Scientific, S21384, 1:100, 40 min). Nuclei were counterstained with Hoechst 33342 (Thermo Fisher Scientific, H3570; 1:100 dilution). Slides were mounted using ProLong™ Gold Antifade Mountant (Thermo Fisher Scientific, P10144) and stored protected from light until image acquisition.

### 2.11 Image acquisition and quantitative analysis

Following multiplexed immunofluorescence, slides were scanned using the Zeiss Axio Scan Z1 slide scanner (Carl Zeiss Microscopy GmbH). Fluorescence images were acquired using DAPI, AF488, and Cy3 channels with 0.75 ms, 10 ms, and 12 ms of exposure time, respectively, and exported for quantitative image analysis. Image registration, fusion and quantitative analysis were performed using HALO® AI v4.1.3 and HALO® image analysis software (Indica Labs). Tumour epithelial cells identified by AE1/AE3 staining were used as the analytical population for quantification of BACH1 expression.

HALO AI™ was used to develop a customised nuclear segmentation classifier based on deep-learning neural network algorithms. This classifier was trained to minimise false-positive nuclear segmentation caused by autofluorescence commonly observed in FFPE tissue sections. The High Plex FL (v4.1.3) module within HALO® was subsequently used to quantify BACH1 fluorescence intensity within the segmented nuclear and cytoplasmic compartments of AE1/AE3-positive tumour cells. An intensity threshold of 5000 was established during assay optimisation based on visual inspection of staining patterns and background fluorescence levels across representative tissue sections. This threshold was selected to minimise the inclusion of background signal and autofluorescence while retaining specific BACH1 staining and was subsequently applied uniformly to all samples. Cells were classified as BACH1^High^ when compartment-specific intensity exceeded this threshold and BACH1^Low^ when intensity was ≤5000. To investigate the relationship between nuclear and cytoplasmic BACH1 expression, two-dimensional scatter plots were generated. Individual cells from the complete cohort were classified into four phenotypic groups according to nuclear and cytoplasmic BACH1 expression status (low/low, high/low, low/high, and high/high). The proportion of cells within each category was quantified to characterise intratumoural phenotypic heterogeneity. The percentage of cells corresponding to each phenotype was exported for downstream analysis.

For each patient, quantitative measurements were obtained from all available TMA cores. Phenotype percentages were calculated independently for each core and subsequently averaged across the eight cores to generate a single representative patient-level value for statistical analysis.

Clinically relevant cut-offs were determined using X-tile software, which identifies thresholds that optimally separate patient groups with favourable and poor clinical outcomes. The final thresholds selected for analysis were 1.5% for compartment-specific BACH1^High^ phenotypes and 5% for total BACH1^High^ levels.

All statistical analyses were performed using Python. Quantitative outputs generated by HALO® and HALO AI™ were exported for downstream analysis. Associations between BACH1-defined phenotypes and patient survival were assessed using Kaplan–Meier survival analysis. Hazard ratios (HRs) and 95% confidence intervals (CIs) were calculated using univariable Cox proportional hazards regression models. All statistical tests were two-sided, and statistical significance was defined as p < 0.05.

### 2.12 Internal validation by bootstrapping

Five-year overall survival was analysed using survival time in months and death status, with follow-up administratively censored at 60 months. Four binary BACH1 phenotypes were evaluated separately using univariable Cox proportional-hazards regression in the 174-patient cohort. The phenotypes nuclear BACH1 High/cytoplasmic BACH1 Low, nuclear BACH1 Low/cytoplasmic BACH1 High, and nuclear BACH1 High/cytoplasmic BACH1 High were classified as positive when more than 1.5% of tumour cells exhibited the corresponding phenotype, whereas n/c BACH1 High was classified as positive when expression exceeded 5%. The phenotype-negative category was used as the reference group. One missing n/c BACH1 High value was confirmed to correspond to a phenotype-negative case and was therefore coded as 0 before analysis.

Internal validation and assessment of sampling variability were performed using non-parametric patient-level bootstrapping with 2000 repetitions for each phenotype. In each repetition, 174 observations were sampled with replacement from the original cohort of 174 patients. Consequently, individual patients could be selected multiple times, whereas others could be absent from a given bootstrap sample. Each bootstrap sample therefore contained 174 observations, corresponding to a median of 110 distinct patients and 64 repeat selections of patients who had already been sampled. The complete patient record, including BACH1 phenotype, survival time, and event status, was retained each time a patient was selected. The corresponding Cox proportional-hazards model was refitted independently in each bootstrap sample, with tied event times handled using Efron's method. Hazard ratios (HRs) were calculated as exp(β), where β is the Cox regression coefficient. Conventional 95% confidence intervals (CIs) and two-sided P values for the original cohort analyses were calculated using the Wald method.

Bootstrap estimates were summarised using the median HR and two-sided 95% bias-corrected and accelerated (BCa) confidence intervals. BCa confidence intervals were calculated on the log-HR scale using leave-one-patient-out jackknife estimates and subsequently exponentiated. Directional stability was defined as the proportion of bootstrap coefficients with the same direction as the corresponding coefficient from the original cohort. Benjamini–Hochberg correction was applied across the four original Cox regression models.

Analyses were performed using a custom Python script in Python 3.12.13 with NumPy 2.3.5, pandas 2.2.3, and SciPy 1.17.0. A fixed random-number seed (20260818) was used to ensure reproducibility.

## 3 Results

### 3.1 Selection and initial screening of commercial anti-BACH1 antibodies

To identify reliable tools for the study of BACH1, we first performed a comprehensive evaluation of commercially available anti-human BACH1 antibodies. Antibodies were selected based on their prior use in key applications, western blotting (WB), immunofluorescence (IF), and immunohistochemistry (IHC), and on citation frequency in peer-reviewed studies. Additional criteria included the availability of detailed immunogen information, reported validation data, and vendor reliability. From this analysis, we assembled a panel of five BACH1 antibodies representing the main commercial sources and a range of immunogens spanning distinct BACH1 domains (Fig. 1a and b).

Although the predicted molecular weight for BACH1 is 82 kDa, its observed mobility is usually observed around 100-110 kDa. However, some studies reported Western blot bands below 100 kDa and in some cases around 70 kDa, often without definitive validation of band identity. To address this variability, we systematically evaluated five BACH1 antibodies by Western blot using multiple validation systems, including wild-type and BACH1 knockout cell lines in three lung cancer cell lines (H1944, A549 and H1299) (Fig. 2a, Suppl Fig. S2a ), as well as cells transfected with control or BACH1-targeting siRNA (Suppl. Fig. S2b). These experiments allowed us to distinguish full-length BACH1 signal from non-specific bands and from potential BACH1 isoforms or cleavage/degradation products and highlighted potential cell line- and antibody-specific differences. By using both complete knockout clones as well as transient knockdowns, we reduced the risk of having CRISPR-mediated or clone-specific effects. All antibodies were initially tested under standard lysis and denaturing conditions (RIPA buffer plus sonication) and optimised working concentrations were established individually for each antibody: Santa Cruz 1:1000, ATLAS 1:1000, Proteintech 1:15000, Bethyl 1:7000, R&D Systems 1:7000.

**Fig. 2:**
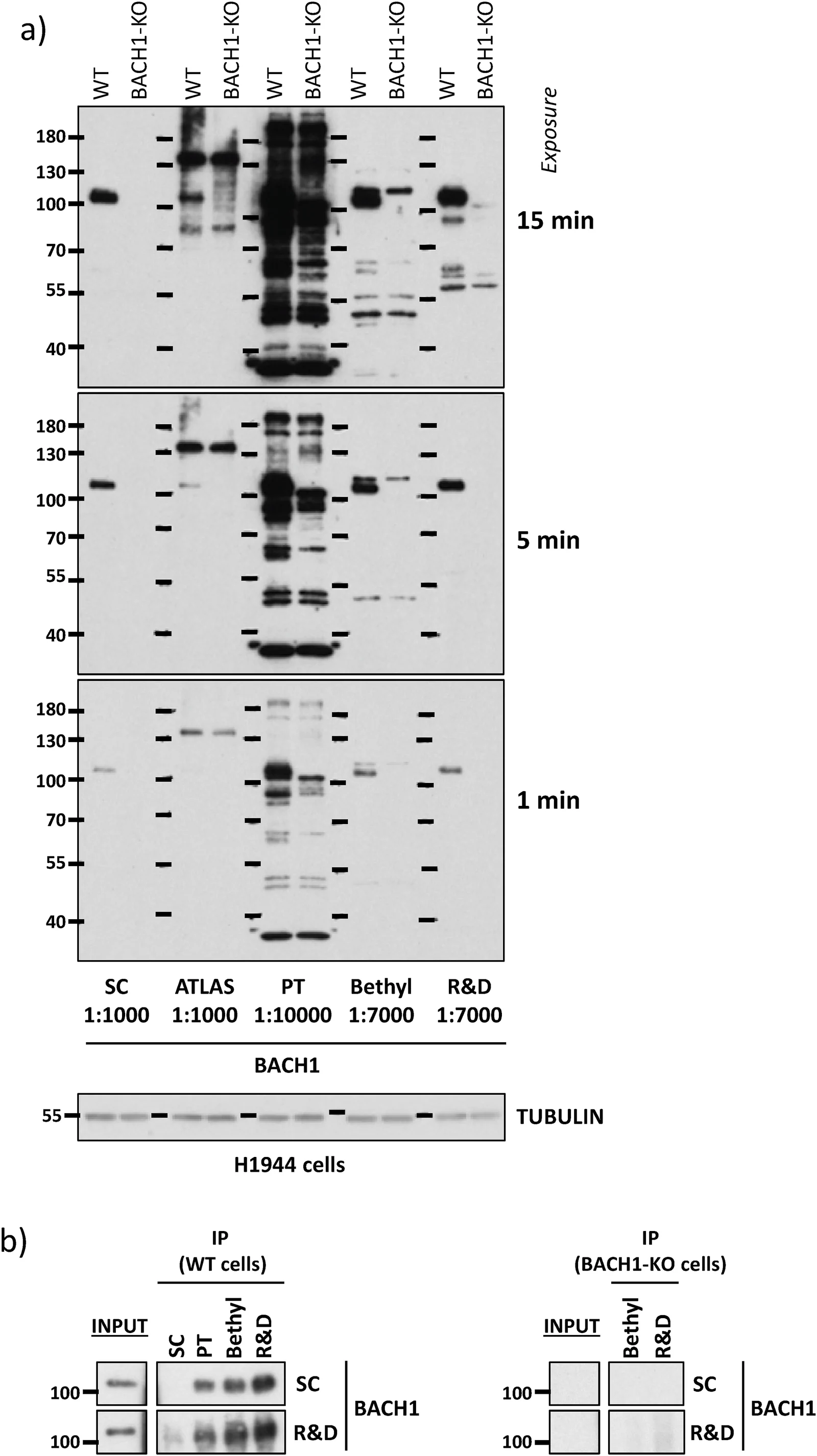
**Characterisation of BACH1 antibodies**. **a)** Equal numbers of H1944 control (WT) and BACH1-knockout (KO) cells were lysed and analysed by western blotting for BACH1 levels using the indicated antibodies. Full blots and exposure times are shown. **b)** Lysates from H1299 WT (left) and BACH1-KO (right) cells were incubated with the indicated BACH1 antibodies and protein G magnetic beads. Following washing and elution, immunoprecipitated proteins were analysed by Western blot using the Santa Cruz (SC) and R&D Systems (R&D) BACH1 antibodies, as indicated. The input (INPUT) represents 10% of the total lysate used.

All antibodies detected a band consistent with full-length BACH1 (∼110 kDa) that was present in WT/siC cells and largely absent in BACH1-KO and siBACH1 cells across most cell lines, supporting the identification of this band as the canonical BACH1 species (Fig. 2a). The only exception was the ATLAS antibody, which did not detect a clear band in A549 cells (Suppl. Fig. S2a and b). Among the antibodies tested, the Santa Cruz (SC) and R&D Systems (R&D) antibodies produced the most defined patterns, detecting fewer bands under our experimental conditions. In contrast, the ATLAS, Proteintech (PT), and Bethyl antibodies generated multiple additional bands. While this pattern is consistent with non-specific cross-reactivity, it is also possible that some of these signals represent alternative BACH1 isoforms, post-translationally modified forms, or partial degradation products. In fact, several lower molecular weight bands detected by some antibodies were also reduced upon BACH1 depletion (Fig. 2a and Suppl. Fig. S2a and b), suggesting that they may represent BACH1-related species rather than non-specific signals. The presence of such species may contribute to the variability in reported BACH1 molecular weights in the literature and complicate the interpretation of Western blot results when appropriate validation controls are not included. Notably, the Proteintech and Bethyl antibodies detected bands migrating very close to the expected BACH1 molecular weight that persisted in knockout or knockdown cells. This persistence suggests that these bands are unlikely to correspond to BACH1.

To further assess band identity and evaluate the suitability of the antibodies for immunoprecipitation (IP), we performed IP experiments using all BACH1 antibodies (except ATLAS which was excluded due to low concentration of the available stock). Immunoprecipitates were then analysed by Western blot using the two best-performing antibodies from the previous analysis (SC and R&D), and as these antibodies recognize non-overlapping regions of BACH1, their combined use provides an additional layer of confidence in specificity. With the exception of the SC antibody, all other antibodies tested (PT, Bethyl, and R&D) efficiently immunoprecipitated BACH1 (Fig. 2b, left panel) while no bands were detected in BACH1-KO cells (Fig. 2b, right panel).

### 3.2 Validation of antibody performance in western blotting across multiple conditions

#### *Validation using functional BACH1 inhibitors*

To further validate the identity of the identified 110 kDa band and to test whether the antibodies accurately reflect changes in BACH1 abundance upon pharmacological modulation, we treated cells with two compounds known to modulate BACH1 through distinct mechanisms of action. Hemin, a heme-derived compound that reduces total BACH1 levels [25], and the electrophilic triterpenoid CDDO-TFEA, which drives BACH1 nuclear export and cytoplasmic accumulation without decreasing total protein levels [22]. As shown in Fig. 3a, hemin treatment resulted in a reduction of BACH1 whole-cell lysate signal intensity across the panel of antibodies, although for two of the antibodies (PT and Bethyl) the reduction was less apparent, particularly at longer exposures. On the other hand, exposure to CDDO-TFEA (Fig. 3b) led to a clear reduction in the nuclear levels of BACH1 with concomitant cytoplasmic accumulation for all antibodies tested.

**Fig. 3:**
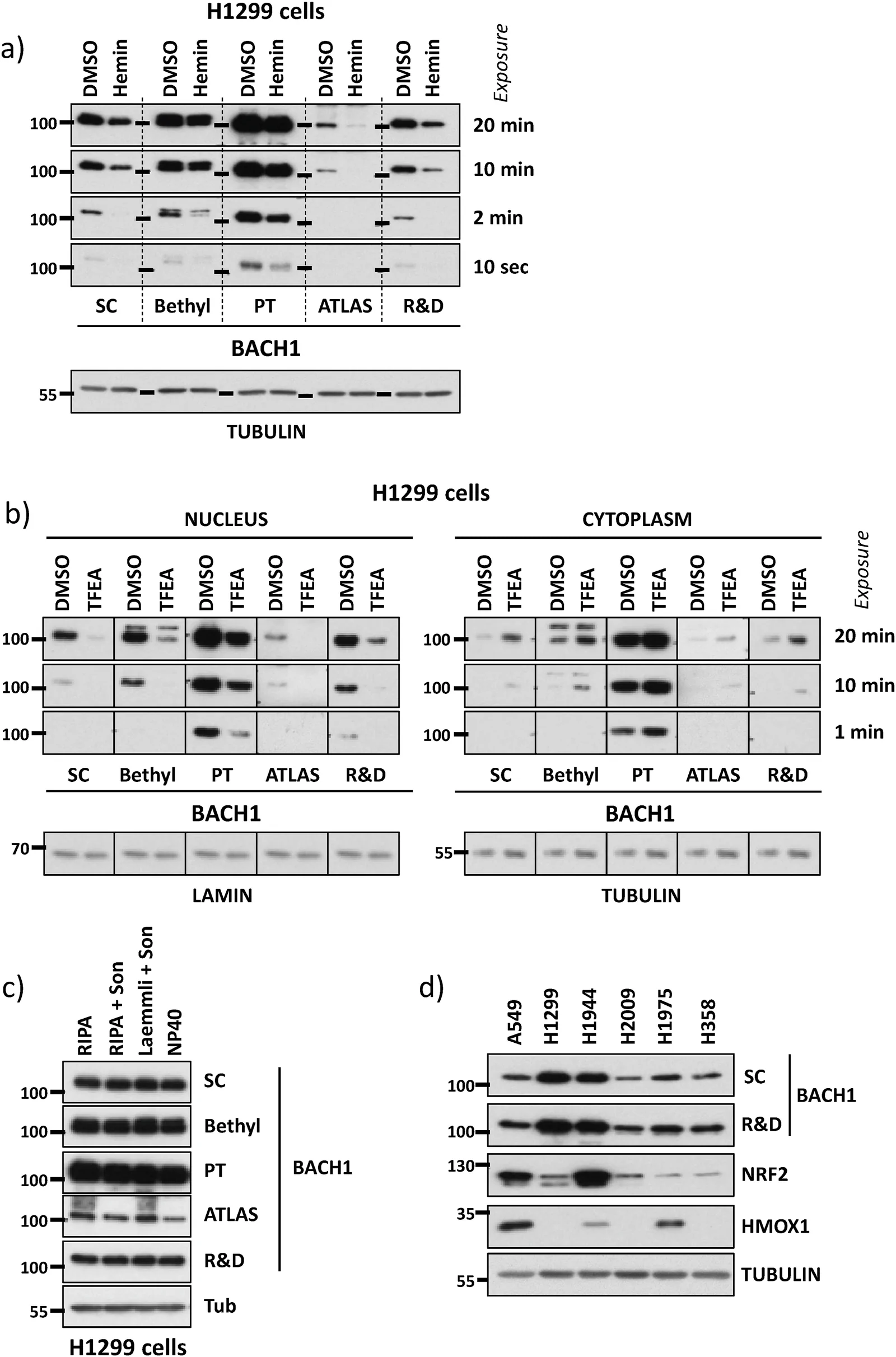
**Assessment of BACH1 antibody performance under pharmacological perturbations, varying lysis conditions, and across different cell types**. **a)** H1299 cells were incubated with DMSO or hemin (15 μM). Five hours later cells were lysed and the levels of total BACH1 were analysed by Western blot using the indicated antibodies. **b)** H1299 cells were treated with DMSO or CDDO-TFEA (100 nM). After 5 h, cells were lysed and fractionated into nuclear and cytoplasmic components. BACH1 levels in each fraction were analysed by Western blot using the indicated antibodies. LAMIN and TUBULIN were used as nuclear and cytoplasmic markers, respectively. All antibodies, but the ATLAS, were developed in the same gel. **c)** Equal amounts of H1299 cells were lysed with various buffers with or without sonication as indicated. The levels of BACH1 were analysed by Western blot using the indicated antibodies. **d)** Equal amount of a panel of lung adenocarcinoma cell lines were lysed and the levels of BACH1 were tested by Western blot using the indicated antibodies. Antibodies against NRF2 and HMOX1 were included for information.

#### *Validation across multiple lysisconditions*

As the ATLAS antibody showed poor performance under some extraction conditions, we next evaluated the robustness and reliability of each antibody for western blotting across multiple lysis and extraction protocols. This approach ensured that the selected antibodies consistently recognize BACH1 under different experimental conditions and improves confidence in reproducibility across laboratories using distinct extraction methods. To this end, we tested a panel of lysis buffers that differed in detergent composition (lysis strength) and mechanical disruption. Specifically, we compared protein extraction using the following conditions:

- RIPA buffer, a widely used lysis buffer containing ionic and non-ionic detergents (SDS, Sodium Deoxycholate, Triton X-100/NP-40), capable of solubilizing cytoplasmic and nuclear proteins causing significant protein denaturation. RIPA extraction was tested both with and without sonication to evaluate whether mechanical disruption improves recovery of chromatin-associated BACH1.
- Laemmli buffer (SDS-based) combined with sonication representing a highly stringent condition that ensures complete protein denaturation and maximal solubilization of nuclear and chromatin-bound proteins. This condition allowed us to assess whether antibody recognition of BACH1 depends on epitope conformation or is preserved under fully denaturing conditions.
- NP-40 buffer, a milder non-denaturing lysis system containing the non-ionic detergent NP-40, which preferentially extracts cytosolic and loosely associated nuclear proteins. This condition was included to evaluate antibody performance under gentler extraction conditions that better preserve protein structure.

As shown in Fig. 3c, all antibodies consistently detected endogenous BACH1 across the lysis conditions tested. These results suggest that, in the analysed cell type, BACH1 is predominantly present in a soluble fraction, as mild NP-40 extraction was sufficient to recover most of the protein.

#### *Evaluation across electrophoretic conditions*

To investigate whether differences in electrophoretic conditions could contribute to variation in the apparent molecular weight of BACH1 that has been reported in different studies, we analysed the same lysates using different gel and buffer systems. Under our standard conditions using 8% or 10% Tris-glycine gels, BACH1 consistently migrated at an apparent molecular weight of approximately 100-110 kDa. In contrast, when the same samples were analysed using pre-casted 4-12% Bis-Tris gels with MOPS running buffer, the BACH1 band migrated at approximately 85-90 kDa (Suppl. Fig. S3a). Thus, the apparent electrophoretic mobility of BACH1 was influenced by the type of gel and buffer system used, with the same protein sample displaying a difference of approximately 15-20 kDa in apparent molecular weight, a phenomenon observed for other proteins, including NRF2.

#### *Evaluation across a panel of lung cancer cell lines*

Among the antibodies tested and across the extraction conditions evaluated, the Santa Cruz and R&D antibodies showed the most consistent and robust performance. Assessing antibody performance across cell lines is an important step to ensure that detection is reliable and accurately reflects endogenous variation in BACH1 expression across biologically relevant genetic and phenotypic contexts. To address this, we used the two selected antibodies to evaluate BACH1 levels across a diverse panel of non-small cell lung cancer (NSCLC) cell lines (Fig. 3d). Both antibodies revealed variable total BACH1 levels among cell lines, reflecting intrinsic differences in BACH1 expression. Notably, despite recognising non-overlapping regions of the protein (see Fig. 1), both yielded consistent expression patterns across the panel, reinforcing the reliability and specificity of the observed signals.

Collectively, these results demonstrate that the selected antibodies (SC and R&D) reliably detect endogenous BACH1 by western blotting across multiple cell types, lysis conditions, and pharmacological perturbations, establishing them as robust tools for quantifying BACH1 levels and modulation by western blotting techniques in cell models.

### 3.3 Optimisation and validation in immunohistochemistry (IHC)

In western blots proteins are denatured and linearized, allowing antibodies to recognize linear epitopes, whereas in other techniques like immunohistochemistry (IHC) or immunofluorescence (IF) the proteins remain in a more native conformation, exposing different three-dimensional or post-translationally modified epitopes within fixed and crosslinked tissues. As a result, an antibody that performs well in WB may fail to detect its target or show altered subcellular localisation patterns in IHC or IF.

To establish a robust and reproducible protocol for BACH1 detection in formalin-fixed, paraffin-embedded tissue samples (FFPE), as required for both tissue IHC and tissue IF, we next evaluated the panel of antibodies for IHC applications. We performed the initial validation using FFPE pellets generated from H1299 wild-type (WT) and pooled BACH1-knockout (KO) cells, which served as matched positive and negative controls. Under these conditions, only the Santa Cruz and the ATLAS antibodies produced a clear differential staining between WT and KO pellets, with a strong, predominantly nuclear signal in WT cells and markedly reduced or absent staining in the KO controls (Fig. 4a). These two antibodies were therefore selected for further optimisation and testing in tissue sections.

**Fig. 4:**
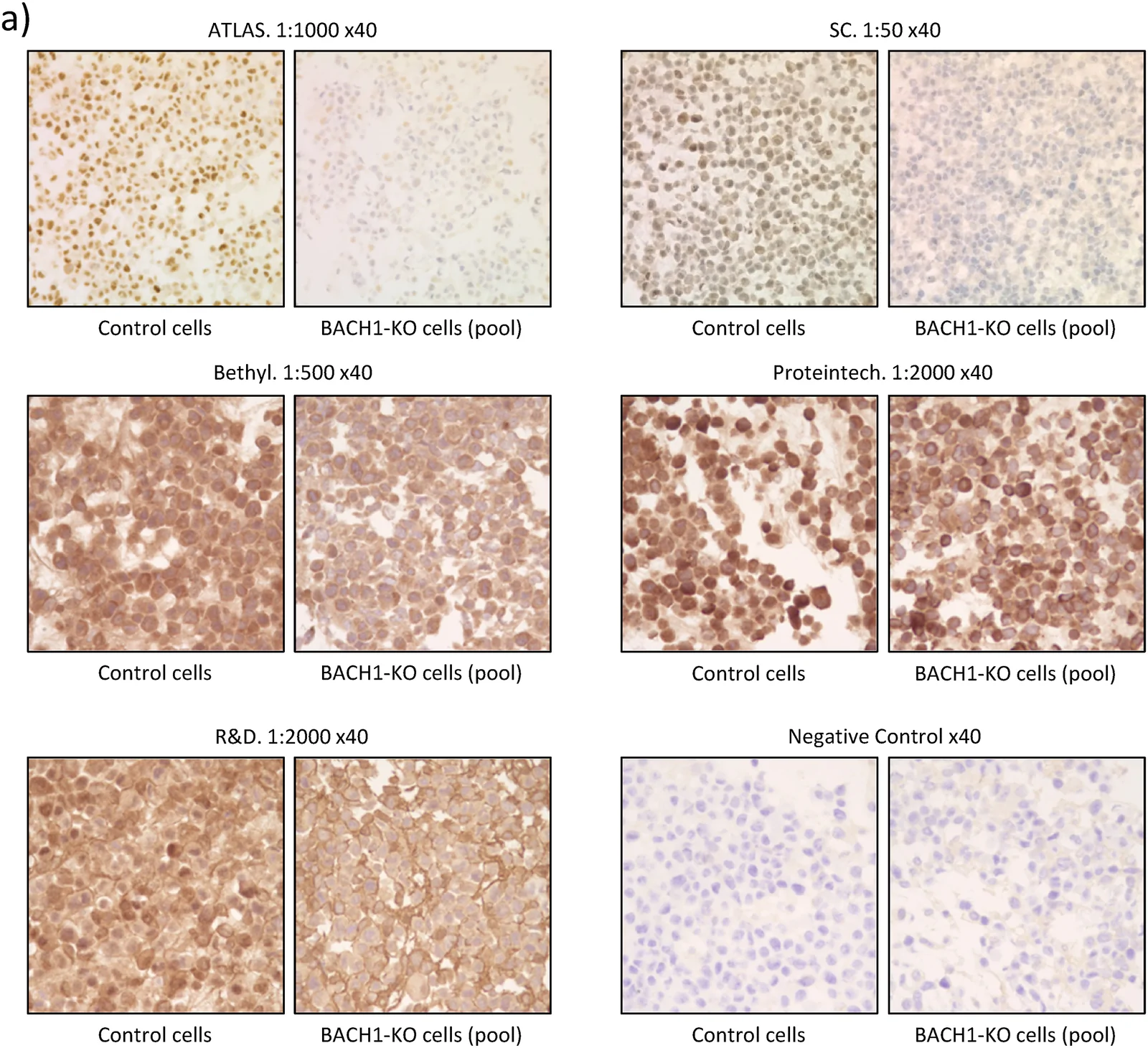
**Validation of BACH1 antibodies for IHC. a)** 40× magnification images comparing staining of formalin-fixed, paraffin-embedded cell pellets from WT and BACH1-KO H1299 cells using the indicated antibodies.

### 3.4 Application of validated antibodies in human tissues

To confirm the capacity of the validated antibodies to detect BACH1 in fixed human tissue samples, we performed a side-by-side comparison of the Santa Cruz and ATLAS BACH1 antibodies using a panel of human tissues that, based on the Human Protein Atlas database (available at proteinatlas.org), show either relatively high levels (skin, tonsils and cervix) or low levels of BACH1 expression (cerebellum and kidney). To enable direct comparison, sequential tissue sections were stained under identical conditions. Both antibodies produced staining patterns consistent with BACH1 expression (Fig. 5a), while the negative control did not show any signal (Suppl. Fig. S5a), supporting the suitability of the antibodies for IHC in human tissues.

**Fig. 5:**
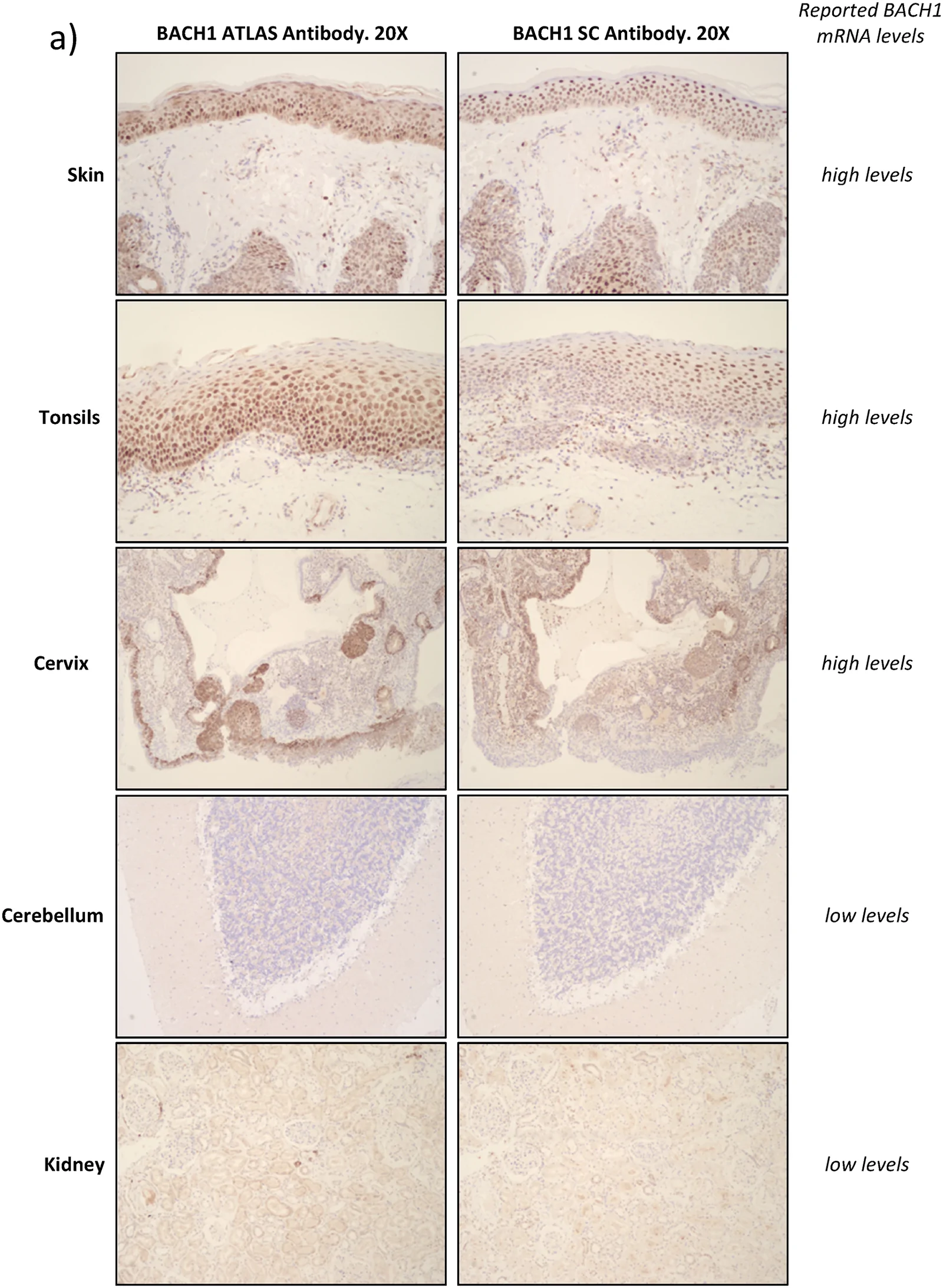
**Side-by-side comparison of BACH1 antibodies in human tissue. a)** Representative 20× magnification images of selected human tissues stained with the indicated BACH1 antibodies. Reported levels of BACH1 expression from Human Protein Atlas.org are shown for reference.

Based on these findings and as the ATLAS antibody it has documented IHC validation in human tissues and produced a staining pattern consistent with the expected nuclear localisation of BACH1, it was selected for extensive validation across a tissue microarray (TMA) comprising lung cancer samples. The staining revealed clear inter-tumoural heterogeneity, with samples showing absent (Suppl. Fig. S5b), exclusively nuclear (Suppl. Fig. S5c), exclusively cytoplasmic (Suppl. Fig. S5d), or mixed (Suppl. Fig. S5e) staining patterns. This variability suggests potential differences in BACH1 localisation among tumour types and individual patients.

### 3.5 Correlation of BACH1 levels and localisation with clinical outcome in early-stage lung adenocarcinoma

Our data show that this protocol allows detection of differences not only in BACH1 levels but also in its subcellular localisation, a feature that is particularly important for the analysis of BACH1 in tissue samples.

BACH1 is a transcriptional repressor whose activity depends on its nuclear localisation. Indeed, we have shown that cytoplasmic BACH1 fails to repress canonical target genes [22]. Consequently, measuring total BACH1 levels without distinguishing between nuclear and cytoplasmic pools may lead to incomplete or potentially misleading interpretations of its biological and clinical significance, however, most studies in human tissue have focused primarily on BACH1 total protein abundance. These considerations highlight the importance of validated tools capable of accurately resolving BACH1 localisation in tissue samples.

Our comprehensive IHC validation ensured that the selected antibodies were reliable in FFPE material, and suitable for assessing both BACH1 abundance and localisation in situ, which was essential before applying them to more complex staining platforms. With these optimised reagents and protocols in place, we then used multiplexed immunofluorescence (mIF) to analyse BACH1 within a unique TMA comprising samples from stage I and II lung adenocarcinoma patients with clinical follow-up information. Multiplexed immunofluorescence allowed for quantitative, single-cell measurement of BACH1 intensity within defined nuclear and cytoplasmic compartments while simultaneously identifying tumour epithelial cells using cytokeratin staining; this provides greater spatial resolution and objectivity than conventional IHC for assessing BACH1 subcellular localisation.

Tumour samples were obtained from patients with early-stage non–small cell lung cancer (NSCLC) who underwent primary surgical resection. Eligible cases included individuals with stage I-IIA/IIB adenocarcinoma, and a five-year survival cut-off was applied for outcome analyses. Because eligible cases were stage I–IIA/IIB and underwent complete resection without neoadjuvant therapy, baseline heterogeneity was reduced. Quantitative single-cell analysis of BACH1 expression across nuclear and cytoplasmic compartments in 174 patients further confirmed this intratumoural heterogeneity in this cohort (Fig. 6a). Fluorescence intensity values were normalised against positive and negative controls, where the BACH1 signal reached approximately 5000 fluorescence intensity units. This threshold was applied to define BACH1^Low^ (≤5000) and BACH1^High^ (>5000). Based on this classification, the majority of tumour cells (94.45%) exhibited BACH1^Low^ levels in both compartments (Q1), while smaller subsets showed BACH1^High^ enrichment restricted to either the cytoplasm (Q2, 1.53%) or nucleus (Q3, 1.96%). A minority of cells (Q4, 2.06%) displayed concurrent high expression in both nuclear and cytoplasmic compartments (Fig. 6b).

**Fig. 6:**
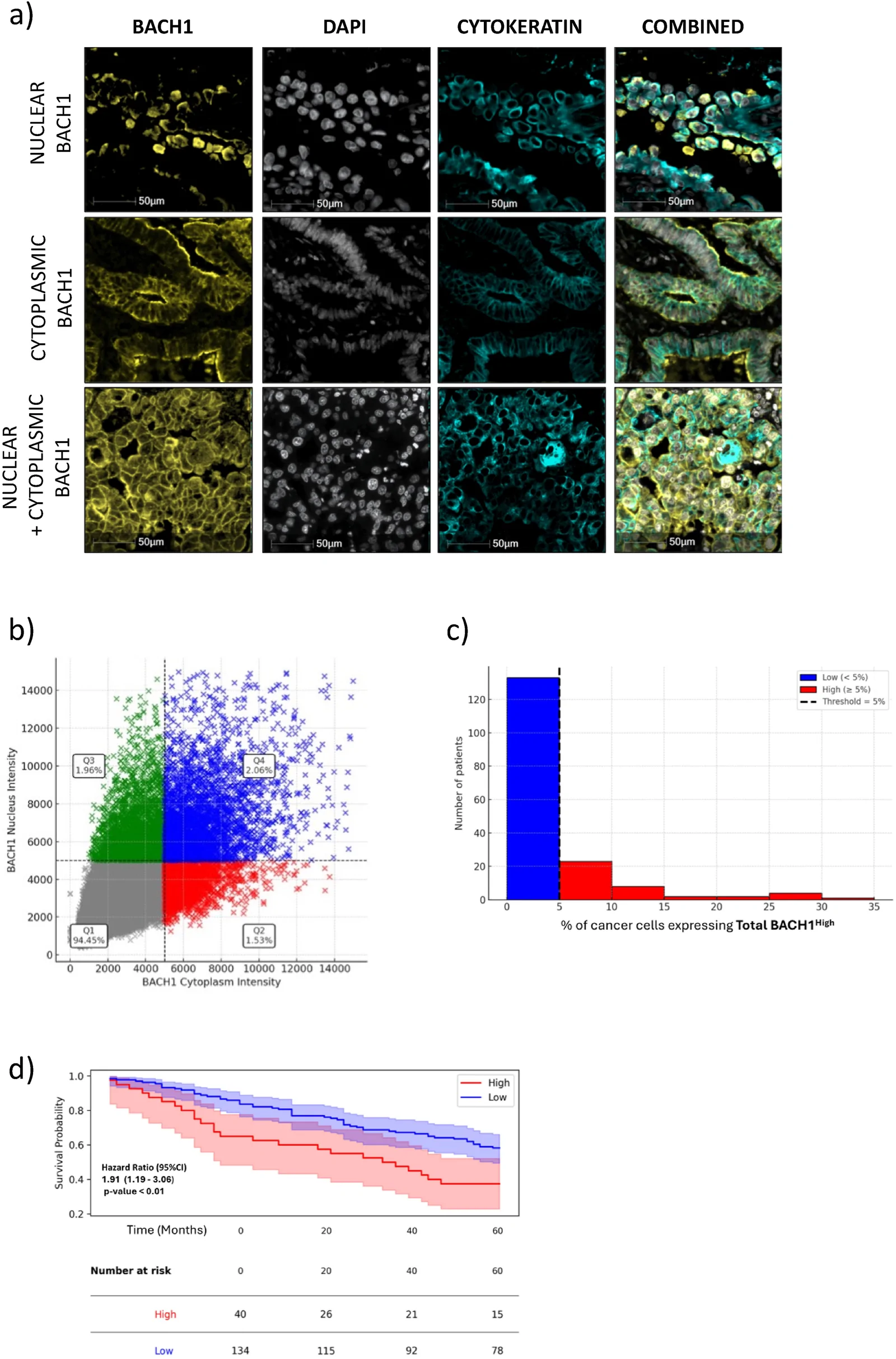
**BACH1 distribution, levels, and prognostic associations. a)** Representative multiplex immunofluorescence images showing nuclear, cytoplasmic, and combined nuclear-cytoplasmic BACH1 localisation. Scale bars = 50 μm. **b)** Scatter plot of nuclear versus cytoplasmic BACH1 intensity with quadrant analysis (Q1–Q4). **c)** Distribution of patients stratified by total BACH1^High^ levels using a 5% threshold. **d)** Kaplan–Meier analysis showing poorer overall survival in patients with high total BACH1^High^ levels. Shaded areas indicate 95% confidence intervals.

Analysis of BACH1^High^ expression across lung cancer tissues revealed clear interpatient variability. Quantification of BACH1^High^ tumour cells demonstrated that most patients (134 of 174 patients, 77%) had low BACH1^High^ levels, defined as fewer than 5% of tumour cells expressing BACH1^High^. In contrast, a clinically relevant subgroup (40 of 174 patients, 23%) displayed high levels of BACH1^High^, defined as more than 5% of tumour cells expressing BACH1^High^ (Fig. 6c). This 5% threshold was not selected arbitrarily but determined using X-tile software, which identifies the most statistically significant cut-off by optimising the relationship between biomarker expression and patient outcome. This data-driven stratification therefore provided an exploratory framework for evaluating the prognostic relevance of BACH1 expression.

Kaplan-Meier survival analysis confirmed the clinical significance of this classification, as patients with more than 5% of tumour cells expressing BACH1^High^, had significantly worse overall survival compared with those below this threshold (HR = 1.91, 95% CI: 1.19–3.06, *P* < 0.01) (Fig. 6d). Collectively, these findings indicate that higher BACH1 levels correlate with adverse prognosis in early-stage lung adenocarcinoma.

To further dissect the prognostic relevance of BACH1^High^, we stratified patients according to nuclear-only BACH1, cytoplasmic-only BACH1, and combined nuclear and cytoplasmic BACH1 levels. For each compartment, a cut-off of 1.5% of tumour cells expressing BACH1^High^ was applied as identified by X-tile. Analysis of nuclear-only BACH1^High^ demonstrated that most patients had low levels of nuclear-only BACH1^High^ (<1.5%), whereas a subset (55 of 174, 32%) showed higher levels of nuclear-only BACH1^High^ (Fig. 7a). Kaplan-Meier survival curves showed that patients with more than 1.5% of cancer cells expressing nuclear-only BACH1^High^ had significantly reduced overall survival compared with those with less than 1.5% nuclear-only BACH1^High^ (HR = 1.86, 95% CI: 1.19-2.90, p < 0.01) (Fig. 7b).

**Fig. 7:**
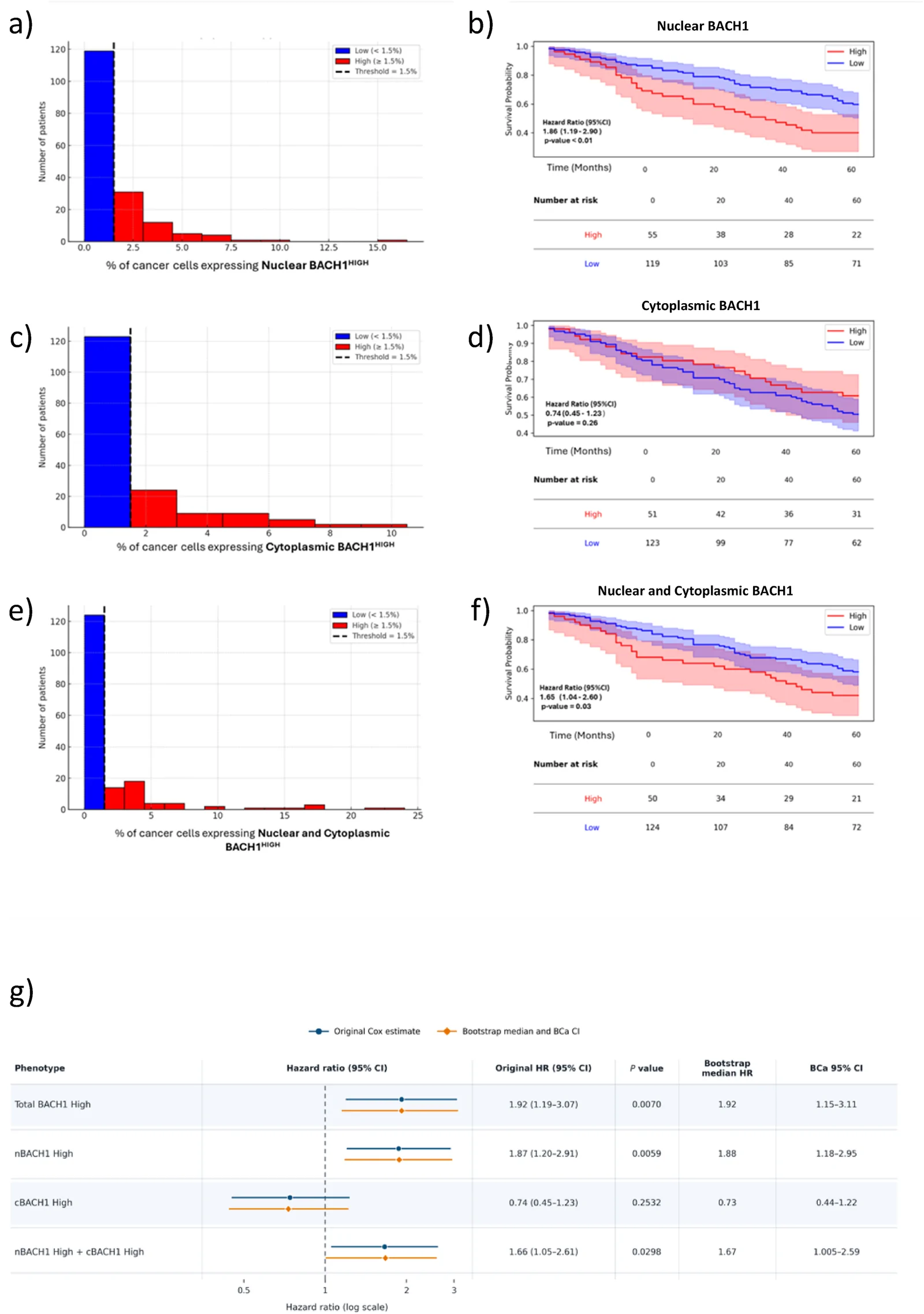
**Prognostic significance of BACH1^High^ localisation.** Histograms show the distribution of patients according to nuclear (**a**), cytoplasmic (**c**), and combined nuclear-cytoplasmic (**e**) BACH1^High^ levels. The dashed line indicates the X-tile-derived cut-off (1.5%) used to define BACH1^High^ and BACH1^Low^ groups. Corresponding Kaplan–Meier curves are shown in (**b**), (**d**), and (**f**). Shaded areas represent 95% confidence intervals. **g**) Forest plot showing hazard ratios (HRs) and 95% confidence intervals (CIs) for overall survival for the original analysis, and the bias-corrected and accelerated (BCa) 95% confidence intervals (CI) after bootstrapping for: total BACH1 high (either nuclear or cytoplasmic high), nBACH1 high (only-nuclear BACH1 high), cBACH1 high (only-cytoplasmic BACH1 high) or nBACH1 + cBACH1 high (both nuclear and cytoplasmic BACH1 high). Points represent HRs and horizontal lines indicate 95% CIs. The dashed line indicates HR = 1.0.

In contrast, cytoplasmic-only BACH1 levels did not show a significant association with survival. While 51 of 174 patients (29%) exhibited cytoplasmic-only BACH1^High^ expression (Fig. 7c), Kaplan-Meier analysis demonstrated no significant difference in survival between the high and low groups expressing cytoplasmic-only BACH1^High^ (HR = 0.74, 95% CI: 0.44-1.23, p = 0.26) (Fig. 7d). These results suggest that cytoplasmic BACH1 alone was not associated with overall survival.

When tumours with combined nuclear and cytoplasmic BACH1^High^ levels were considered, a clear prognostic association was observed. Although only 50 of 174 patients (29%) were classified as having high combined nuclear and cytoplasmic BACH1^High^ levels (Fig. 7e), these patients had significantly poorer overall survival than those in the low-expression group (HR = 1.65, 95% CI: 1.06-2.60, p < 0.05) (Fig. 7f). Univariable Cox proportional hazards regression analysis further supported these findings (Fig. 7g). Consistent with the Kaplan–Meier analyses, total BACH1^High^, nuclear-only BACH1^High^, and combined nuclear and cytoplasmic BACH1^High^ expression were each associated with an increased risk of death, whereas cytoplasmic-only BACH1^High^ expression alone was not. These data indicate that the adverse prognostic association of BACH1^High^ is primarily associated to its nuclear localisation. The association observed for combined nuclear and cytoplasmic BACH1^High^ levels is likely driven, at least in part, by the presence of a nuclear BACH1^High^ fraction, rather than by cytoplasmic BACH1^High^ levels alone.

To assess the robustness of these associations, we performed internal validation using non-parametric patient-level bootstrapping with 2000 repetitions for each phenotype. The bootstrap estimates closely reproduced the effect sizes observed in the original cohort (Fig. 7g). For nuclear-only BACH1^High^, the original Cox regression yielded an HR of 1.87 (95% CI: 1.20–2.91; P = 0.0059), while the bootstrap median HR was 1.88, with a bias-corrected and accelerated, (BCa) 95% confidence intervals (CI) of 1.18–2.95. Similarly, the association for both nuclear and cytoplasmic BACH1^High^ levels was highly stable, with an original HR of 1.66 (95% CI: 1.05–2.61; P = 0.0298) and a bootstrap median HR of 1.67 (BCa 95% CI: 1.005–2.59). Total BACH1^High^ expression showed comparable stability, with an original HR of 1.92 (95% CI: 1.19–3.07; P = 0.0070) and a bootstrap median HR of 1.92 (BCa 95% CI: 1.15–3.11). In contrast, the lack of association observed for cytoplasmic-only BACH1^High^ was also maintained after bootstrapping, with an original HR of 0.74 (95% CI: 0.45–1.23; P = 0.2532) and a bootstrap median HR of 0.73 (BCa 95% CI: 0.44–1.22).

Thus, the bootstrap analysis demonstrated a high degree of stability in both the magnitude and direction of the observed associations. Importantly, the prognostic effect associated with nuclear BACH1^High^ levels was consistently reproduced across resampled datasets, whereas cytoplasmic-only BACH1^High^ remained non-prognostic. Together, these findings provide internal support for the robustness of the association between nuclear BACH1 localisation and adverse outcome in early-stage lung adenocarcinoma, and further emphasise the importance of assessing BACH1 subcellular localisation rather than total protein abundance alone.

## 4 Discussion

Our systematic evaluation of commercially available BACH1 antibodies highlights the importance of rigorous validation for reliable detection across experimental platforms. In Western blot analyses, the combined use of multiple antibodies targeting independent epitopes together with BACH1 knockout controls proved essential for accurately identifying the full-length protein. Several antibodies produced additional bands migrating close to the expected molecular weight of BACH1 that persisted in knockout lysates, demonstrating the limitations and risks of assigning band identity based solely on apparent size. Among the antibodies tested, those from Santa Cruz and R&D Systems showed the most consistent performance and minimal non-specific signal across conditions.

In addition to the canonical ∼110 kDa band, we identified lower molecular weight species that were reproducibly detected by multiple antibodies and were reduced upon genetic or siRNA-mediated depletion of BACH1. These observations suggest that such bands may represent bona fide BACH1-related species, such as alternative isoforms or proteolytic fragments, rather than non-specific signals. The presence of these species may help explain discrepancies in reported BACH1 molecular weights and highlights the complexity of BACH1 regulation.

WB, IHC, and IF interrogate proteins in fundamentally different biochemical states, meaning that antibody performance in one application does not necessarily predict performance in another, as we have shown in our analysis. For this reason, antibodies should be validated independently for each experimental platform.

The antibody validation strategy also informed the development of robust immunohistochemistry and immunofluorescence protocols for assessing BACH1 expression in human tissues. Antibodies recognising distinct, non-overlapping epitopes produced broadly comparable staining patterns, supporting the overall specificity of BACH1 detection. However, moderate differences in subcellular localisation were observed. The Santa Cruz antibody generated a predominantly nuclear signal, whereas the ATLAS antibody exhibited a broader distribution across nuclear and cytoplasmic compartments. These differences may reflect technical factors, including epitope accessibility and sensitivity to antigen retrieval conditions, as well as biological factors such as differential recognition of BACH1 isoforms or post-translationally modified forms.

Applying the optimised workflow to a lung adenocarcinoma tissue microarray revealed that both the abundance and subcellular localisation of BACH1 appear to carry prognostic information. Higher levels of nuclear BACH1, but not cytoplasmic BACH1, were associated with poorer overall survival in early-stage (stage I-IIA/IIB) lung adenocarcinoma patients. Moreover, the association observed in tumours with combined higher nuclear and cytoplasmic BACH1 levels likely reflects the contribution of the nuclear BACH1 fraction, consistent with the role of BACH1 as a transcriptional regulator. This finding is particularly relevant, given the limited availability of prognostic biomarkers in early-stage lung adenocarcinoma, where additional biomarkers are needed to improve risk stratification beyond standard clinicopathological parameters. If confirmed in independent cohorts and multivariable models, nuclear BACH1 may represent a useful biomarker for identifying patients at higher risk of poor outcome who could benefit from closer monitoring or adjuvant therapeutic strategies.

Some limitations should be considered when interpreting these findings. The objective of this study was not to establish BACH1 as a clinically validated biomarker, and therefore it should not be considered a biomarker validation study. Rather, by applying an optimised and rigorously validated protocol, we highlighted BACH1 subcellular localisation as a previously unexplored dimension of its biology with potential prognostic relevance. The consistency of the prognostic associations after bootstrap resampling, particularly for nuclear BACH1^High^ levels, provides additional support for the robustness of this observation. Although bootstrapping demonstrated that the observed hazard ratios were highly stable, this represents internal validation and does not substitute for validation in an independent patient cohort. Additionally, the cut-offs used to define BACH1^High^ tumours were derived within the same cohort and should therefore be considered exploratory. These findings provide proof-of-concept evidence and a rationale for future studies in independent, clinically annotated cohorts to determine whether BACH1 subcellular localisation can be validated as a prognostic marker and to establish reproducible, clinically applicable thresholds.

In addition, although the multiplex immunofluorescence assay enabled sensitive detection of BACH1, it relied on a single antibody. However, the antibody selected for multiplex immunofluorescence was not chosen in isolation but following extensive validation across multiple complementary approaches, including immunohistochemistry, and comparison with an independent antibody recognising a distinct epitope. These orthogonal validation strategies substantially reduce the likelihood that the observed staining reflects antibody cross-reactivity.

As all validation experiments in this study were performed using human cell lines and human tissue samples, caution should be exercised when extrapolating these findings to other species, as differences in BACH1 sequence, post-translational modifications, or protein processing may influence antibody performance and the apparent molecular weight of BACH1. In addition, our comparison of electrophoretic conditions provides experimental evidence that the apparent molecular weight of BACH1 can vary substantially depending on the type of gel and buffer system used, and thus should be considered when comparing Western blot results between studies.

In summary, this study demonstrates that rigorous antibody validation and assay optimisation are critical for accurate assessment of BACH1 expression. By integrating antibody benchmarking with tissue-based assay development, we establish a framework for reliable BACH1 analysis in both experimental and clinical settings. Our findings further suggest that nuclear localisation of BACH1, rather than overall expression alone, may provide clinically relevant prognostic information in early-stage lung adenocarcinoma. Validation in larger, independent cohorts will be necessary to confirm these observations and determine whether nuclear BACH1 can be incorporated into future biomarker-driven clinical decision-making.

## CRediT authorship contribution statement

**Yumna Sharaf:** Investigation. **Donika Klenja-Skudrinja:** Investigation. **Maureen Higgins:** Investigation. **Susan Bray:** Investigation, Methodology. **David Walker:** Investigation. **Hazem Abdullah:** Formal analysis. **In Hwa Um:** Formal analysis, Methodology. **Aileen Sandilands:** Investigation. **Neil Kernohan:** Methodology. **Albena T. Dinkova-Kostova:** Conceptualization, Writing – review & editing. **David J. Harrison:** Funding acquisition, Conceptualization, Writing – review & editing. **Laureano de la Vega:** Conceptualization, Writing – original draft, Funding acquisition, Supervision.

## Declaration of genrative AI

During the preparation of this work, the author(s) used OpenAI's ChatGPT for editing and proofreading the final document. The author(s) reviewed and edited the output as needed and take full responsibility for the content of the published article.

## Funding

This work was supported by Cancer Research UK (C52419/A22869) and Ninewells Cancer Campaign (DKS). For the purpose of open access, the authors have applied a Creative Commons Attribution (CC BY) licence to any Author Accepted Manuscript version arising from this submission.

## Declaration of competing interest

The authors declare that they have no known competing financial interests or personal relationships that could have appeared to influence the work reported in this paper.
